# Use of Unstructured Event-Based Reports for Global Infectious Disease Surveillance

**DOI:** 10.3201/eid1505.081114

**Published:** 2009-05

**Authors:** Mikaela Keller, Michael Blench, Herman Tolentino, Clark C. Freifeld, Kenneth D. Mandl, Abla Mawudeku, Gunther Eysenbach, John S. Brownstein

**Affiliations:** Harvard–Massachusetts Institute of Technology Division of Health Sciences and Technology, Boston, Massachusetts, USA (M. Keller, C.C. Freifeld, K.D. Mandl, J.S. Brownstein); Children’s Hospital Boston, Boston (M. Keller, C.C. Freifeld, K.D. Mandl, J.S. Brownstein); Harvard Medical School, Boston (M. Keller, C.C. Freifeld, K.D. Mandl, J.S. Brownstein); Public Health Agency of Canada, Ottawa, Ontario, Canada (M. Blench, A. Mawudeku); Centers for Disease Control and Prevention, Atlanta, Georgia, USA (H. Tolentino); University of Toronto, Toronto, Ontario, Canada (G. Eysenbach); University Health Network, Toronto (G. Eysenbach)

**Keywords:** Communicable diseases, disease outbreaks, population surveillance, software design, public health, geography, synopsis

## Abstract

Free or low-cost sources of unstructured information, such as Internet news and online discussion sites, provide detailed local and near real-time data on disease outbreaks, even in countries that lack traditional public health surveillance. To improve public health surveillance and, ultimately, interventions, we examined 3 primary systems that process event-based outbreak information: Global Public Health Intelligence Network, HealthMap, and EpiSPIDER. Despite similarities among them, these systems are highly complementary because they monitor different data types, rely on varying levels of automation and human analysis, and distribute distinct information. Future development should focus on linking these systems more closely to public health practitioners in the field and establishing collaborative networks for alert verification and dissemination. Such development would further establish event-based monitoring as an invaluable public health resource that provides critical context and an alternative to traditional indicator-based outbreak reporting.

## Abstract 2

Free or low-cost unstructured reports offer an alternative to traditional indicator-based outbreak reporting.

International travel and movement of goods increasingly facilitates the spread of pathogens across and among nations, enabling pathogens to invade new territories and adapt to new environments and hosts (*1*–*3*). Officials now need to consider worldwide disease outbreaks when determining what potential threats might affect the health and welfare of their nations (*4*). In industrialized countries, unprecedented efforts have built on indicator-based public health surveillance, and monitoring of clinically relevant data sources now provides early indication of outbreaks (*5*). In many countries where public health infrastructure is rudimentary, deteriorating, or nonexistent, efforts to improve the ability to conduct electronic disease surveillance include more robust data collection methods and enhanced analysis capability (*6*,*7*). However, in these parts of the world, basing timely and sensitive reporting of public health threats on conventional surveillance sources remains challenging. Lack of resources and trained public health professionals poses a substantial roadblock (*8*–*10*). Furthermore, reporting emerging infectious diseases has certain constraints, including fear of repercussions on trade and tourism, delays in clearance through multiple levels of government, tendency to err on the conservative side, and inadequately functioning or nonexistent surveillance infrastructure (*11*). Even with the recent enactment of international health regulations in 2005, no guarantee yet exists that broad compliance will be feasible, given the challenges associated with reporting mechanisms and multilateral coordination (*12*).

In many countries, free or low-cost sources of unstructured information, including Internet news and online discussion sites (Figure), could provide detailed local and near real-time data on potential and confirmed disease outbreaks and other public health events (*9*,*10*,*13*–*18*). These event-based informal data sources provide insight into new and ongoing public health challenges in areas that have limited or no public health reporting infrastructure but have the highest risk for emerging diseases (*19*). In fact, event-based informal surveillance now represents a critical source of epidemic intelligence—almost all major outbreaks investigated by the World Health Organization (WHO) are first identified through these informal sources (*9*,*13*).

**Figure:**
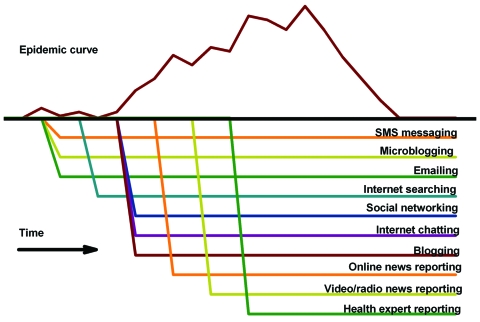
Hypothetical timing of informal electronic sources available during an outbreak. SMS, short message service.

With a goal of improving public health surveillance and, ultimately, intervention efforts, we (the architects, developers, and methodologists for the information systems described herein) reviewed 3 of the primary active systems that process unstructured (free-text), event-based information on disease outbreaks: The Global Public Health Intelligence Network (GPHIN), the HealthMap system, and the EpiSPIDER project (Semantic Processing and Integration of Distributed Electronic Resources for Epidemics [and disasters]; www.epispider.net). Our report is the result of a joint symposium from the American Medical Informatics Association Annual Conference in 2007. Despite key differences, all 3 systems face similar technologic challenges, including 1) topic detection and data acquisition from a high-volume stream of event reports (not all related to disease outbreaks); 2) data characterization, categorization, or information extraction; 3) information formatting and integration with other sources; and 4) information dissemination to clients or, more broadly, to the public.

Each system tackles these challenges in unique ways, highlighting the diversity of possible approaches and public health objectives. Our goal was to draw lessons from these early experiences to advance overall progress in this recently established field of event-based public health surveillance. After summarizing these systems, we compared them within the context of this new surveillance framework and outlined goals for future development and research.

## The GPHIN Project

### Background

GPHIN took early advantage of advancements in communication technologies to provide coordinated, near real-time, multisource, and multilingual information for monitoring emerging public health events (*20*,*21*). In 1997, a prototype GPHIN system was developed in a partnership between the government of Canada and WHO. The objective was to determine the feasibility and effectiveness of using news media sources to continuously gather information about possible disease outbreaks worldwide and to rapidly alert international bodies of such events. The sources included websites, news wires, and local and national newspapers retrieved through news aggregators in English and French. After the outbreak of severe acute respiratory syndrome (SARS), a new, robust, multilingual GPHIN system was developed and was launched November 17, 2004, at the United Nations.

### Data Acquisition

#### Automated process

The GPHIN software application retrieves relevant articles every 15 minutes (24 hours/day, 7 days/week) from news-feed aggregators (Al Bawaba [www.albawaba.com] and Factiva [www. factiva.com]) according to established search queries that are updated regularly. The matching articles are automatically categorized into >1 GPHIN taxonomy categories, which cover the following topics: animal, human, or plant diseases; biologics; natural disasters; chemical incidents; radiologic incidents; and unsafe products.

Articles with a high relevancy score are automatically published on the GPHIN database. The GPHIN database is also augmented with articles obtained manually from open-access web sites. Each day, GPHIN handles ≈4,000 articles. This number drastically increases when events with serious public health implications, such as the finding of melamine in various foods worldwide, are reported.

#### Human Analysis Process

Although the GPHIN computerized processes are essential for the management of information about health threats worldwide, the linguistic, interpretive, and analytical expertise of the GPHIN analysts makes the system successful. Articles with relevancy below the “publish” threshold are presented to a GPHIN analyst, who reviews the article and decides whether to publish it, issue an alert, or dismiss it. Additionally, the GPHIN analyst team conducts more in-depth tasks, including linking events in different regions, identifying trends, and assessing the health risks to populations around the world.

### Data Dissemination

#### Machine Translation

English articles are machine-translated into Arabic, Chinese (simplified and traditional), Farsi, French, Russian, Portuguese, and Spanish. Non-English articles are machine-translated into English. GPHIN has adopted a best-of-breed approach in selecting engines for machine translation. The lexicons associated with the engines are constantly being improved to enhance the quality of the output. As such, the machine-translated outputs are edited by the appropriate GPHIN analysts. The goal is not to obtain a perfect translation but to ensure comprehensibility of the essence of the article.

#### Information Access

Users can view the latest list of published articles or query the database by using both Boolean and translingual metadata search capabilities. In addition, notifications about events that might have serious public health consequences are immediately sent by email to users in the form of an alert.

### Project Results

As an initial assessment of data collected during July 1998 through August 2001, WHO retrospectively verified 578 outbreaks, of which 56% were initially picked up and disseminated by GPHIN (*9*). Outbreaks were reported in 132 countries, demonstrating GPHIN’s capacity to monitor events occurring worldwide, despite the limitation of predominantly English (with some French) media sources.

One of GPHIN’s earliest achievements occurred in December 1998, when the system was the first to provide preliminary information to the public health community about a new strain of influenza in northern People’s Republic of China (*20*). During the SARS outbreak, declared by WHO in March 2003, the GPHIN prototype demonstrated its potential as an early-warning system by detecting and informing the appropriate authorities (e.g., WHO, Public Health Agency of Canada) of an unusual respiratory illness outbreak occurring in Guangdong Province, China, as early as November 27, 2002. GPHIN was further able to continuously monitor and provide information about the number of suspected and probable SARS cases reported worldwide on a near real-time basis. GPHIN’s information was ≈2–3 days ahead of the official WHO report of confirmed and probable cases worldwide.

In addition to outbreak reporting, GPHIN has also provided information that enabled public health officials to track global effects of the outbreak such as worldwide prevention and control measures, concerns of the general public, and economic or political effects. GPHIN is used daily by organizations such as WHO, the US Centers for Disease Control and Prevention (CDC), and the UN Food and Agricultural Organization.

## The HealthMap Project

### Background

Operating since September 2006, HealthMap (*22*,*23*) is an Internet-based system designed to collect and display information about new outbreaks according to geographic location, time, and infectious agent (*24*–*26*). HealthMap thus provides a structure to information flow that would otherwise be overwhelming to the user or obscure important elements of a disease outbreak.

Healthmap.org receives 1,000–10,000 visits/day from around the world. It is cited as a resource on sites of agencies such as the United Nations, National Institute of Allergy and Infectious Diseases, US Food and Drug Administration, and US Department of Agriculture. It has also been featured in mainstream media publications, such as Wired News and Scientific American, indicating the broad utility of such a system that extends beyond public health practice (*24*,*26*). On the basis of usage tracking of HealthMap’s Internet site, we can infer that its most avid users tend to come from government-related domains, including WHO, CDC, European Centre for Disease Prevention and Control, and other national, state, and local bodies worldwide. Although the question of whether this information has been used to initiate action will be part of an in-depth evaluation, we know from informal communications that organizations (ranging from local health departments to such national organizations as the US Department of Health and Human Services and the US Department of Defense) are leveraging the HealthMap data stream for day-to-day surveillance activities. For instance, CDC’s BioPHusion Program incorporates information from multiple data sources, including media reports, surveillance data, and informal reports of disease events and disseminates it to public health leaders to enhance CDC’s awareness of domestic and global health events (*27*).

### Data Acquisition

The system integrates outbreak data from multiple electronic sources, including online news wires (e.g., Google News), Really Simple Syndication (RSS) feeds, expert-curated accounts (e.g., ProMED-mail, a global electronic mailing list that receives and summarizes reports on disease outbreaks) (*18*), multinational surveillance reports (e.g., Eurosurveillance), and validated official alerts (e.g., from WHO). Through this multistream approach, HealthMap casts a unified and comprehensive view of global infectious disease outbreaks in space and time. Fully automated, the system acquires data every hour and uses text mining to characterize the data to determine the disease category and location of the outbreak. Alerts, defined as information on a previously unidentified outbreak, are geocoded to the country scale with province-, state-, or city-level resolution for select countries. Surveillance is conducted in several languages, including English, Spanish, Russian, Chinese, and French. The system is currently being ported to other languages, such as Portuguese and Arabic.

### Data Dissemination

After being collected, the data are aggregated by source, disease, and geographic location and then overlaid on an interactive map for user-friendly access to the original report. HealthMap also addresses the computational challenges of integrating multiple sources of unstructured information by generating meta-alerts, color coded on the basis of the data source’s reliability and report volume. Although information relating to infectious disease outbreaks is collected, not all information has relevance to every user. The system designers are especially concerned with limiting information overload and providing focused news of immediate interest. Thus, after a first categorization step into locations and diseases, a second round of category tags is applied to the articles to improve filtering. The primary tags include 1) breaking news (e.g., a newly discovered outbreak); 2) warning (initial concerns of disease emergence, e.g., in a natural disaster area; 3) follow-up (reference to a past outbreak); 4) background/context (information on disease context, e.g., preparedness planning); and 5) not disease-related (information not relating to any disease [2–5 are filtered from display]). Duplicate reports are also removed by calculating a similarity score based on text and category matching. Finally, in addition to providing mapped content, each alert is linked to a related information window with details on reports of similar content as well as recent reports concerning either the same disease or location and links for further research (e.g., WHO, CDC, and PubMED).

### Project Results

HealthMap processes an average of 133.5 disease alerts/day (95% confidence interval [CI] 124.1–142.8); ≈50% are categorized as breaking news (65.3 reports/day). Looking 30 days back (default display), the system displays >800 breaking news alerts for any given day. From October 2006 through November 20, 2007, HealthMap had processed >35,749 alerts across 171 disease categories and 202 countries or semiautonomous or overseas territories. Most alerts come from news media (92.8%), followed by ProMED (6.5%) and multinational agencies (0.7%).

## The EpiSPIDER Project

### Background

The EpiSPIDER project was designed in January 2006 to serve as a visualization supplement to the ProMED-mail reports. Through use of publicly available software, EpiSPIDER was able to display topic intensity of ProMED-mail reports on a map. Additonally, EpiSPIDER automatically converted the topic and location information of the reports into RSS feeds. Usage tracking showed, initially, that the RSS feeds were more popular than the maps. Transforming reports to a semantic online format (W3C Semantic Web) makes it possible to combine emerging infectious disease content with similarly transformed information from other Internet sites such as the Global Disaster Alert Coordinating System (GDACS) website (www.gdacs.org). The broad effects of disasters often increase illness and death from communicable diseases, particularly where resources for healthcare infrastructure have been lacking (*28*,*29*). By merging these 2 online media sources (ProMED-mail and GDACS), EpiSPIDER demonstrates how distributed, event-based, unstructured media sources can be integrated to complement situational awareness for disease surveillance.

### Data Acquisition and Dissemination

EpiSPIDER connects to news sites and uses natural language processing to transform free-text content into structured information that can be stored in a relational database. For ProMED reports, the following fields are extracted: date of publication; list of locations (country, province, or city) mentioned in the report; and topic. EpiSPIDER parses location names from these reports and georeferences them using the georeferencing services of Yahoo Maps (http://maps.yahoo.com), Google Maps (http://maps.google.com), and Geonames (www.geonames.org).

Each news report that has location information can be linked to relevant demographic- and health-specific information (e.g., population, per capita gross domestic product, public health expenditure, and physicians/1,000 population). EpiSPIDER extracts this information from the Central Intelligence Agency (CIA) Factbook (www.cia.gov/library/publications/the-world-factbook/index.html) and the United Nations Development Human Development Report (http://hdr.undp.org/en) Internet sites. This feature provides different contexts for viewing emerging infectious disease information. By using askMEDLINE (*30*), EpiSPIDER also provides context-sensitive links to recent and relevant scientific literature for each ProMED-mail report topic. After EpiSPIDER extracts the previously described information, it automatically transforms it to other formats, e.g., RSS, keyhole markup language (KML; http://earth.google.com/kml), and JavaScript object notation (JSON, a human-readable format for representing simple data structures; www.json.org). Publishing content using those formats enables the semantic linking of ProMED-mail content to country information and facilitates EpiSPIDER’s redistribution of structured data to services that can consume them. Continuing along this transformation chain, the SIMILE Exhibit API (http://simile.mit.edu) that consumes JSON-formatted data files enables faceted browsing of information by using scatter plots, Google Maps, and timelines.

Recently, EpiSPIDER began outsourcing some of its preprocessing and natural language processing tasks to external service providers such as OpenCalais (www.opencalais.com) and the Unified Medical Language System (UMLS) web service for concept annotation. This action has enabled the screening of noncurated news sources as well.

### Project Results

Built on open-source software components, EpiSPIDER has been operational since January 2006. In response to feedback from users, additional custom data feeds have been incorporated, both topic oriented (by disease) and format specific (KML, RSS, GeoRSS), as has semantic annotation using UMLS concept codes. For example, the EpiSPIDER KML module was developed to enable the US Directorate for National Intelligence to distribute avian influenza event-based reports in Google Earth KML format to consumers worldwide and also to enable an integrated view of ProMED and World Animal Health Information Database reports.

EpiSPIDER is used by persons in North America, Europe, Australia, and Asia, and it receives 50–90 visits/hour, originating from 150–200 sites and representing 30–50 countries worldwide. EpiSPIDER has recorded daily visits from the US Department of Agriculture, US Department of Homeland Security, US Directorate for National Intelligence, US CDC, UK Health Protection Agency, and several universities and health research organizations. In the latter half of 2008, daily access to graphs and exhibits surpassed access to data feeds. EpiSPIDER’s semantically linked data were also used for validating syndromic surveillance information in OpenRODS (http://openrods.sourceforge.net) and populating disease detection portals, like www.intelink.gov and the Research Triangle Institute (Research Triangle Park, NC, USA).

## Discussion

Despite their similarities, the 3 described event-based public health surveillance systems are highly complementary; they monitor different data types, rely on varying levels of automation and human analysis, and distribute distinct information. GPHIN, being the longest in use, is probably the most mature in terms of information extraction. In contrast, HealthMap and EpiSPIDER, being comparatively recent programs, focus on providing extra structure and automation to the information extracted. Their differences and similarities, summarized in the Table, can be analyzed according to multiple characteristics: What data sources do they consider? How do they extract information from those sources? And in what format is the information redistributed and how?

**Table:** Characteristics of 3 primary systems that process event-based informal data sources*

System	Data sources (languages)	Data characterization	Information formatting	Data dissemination		
				Access	User interface	Format
GPHIN	Factiva, Al Bawaba (9 languages)	Automatic and human	Categorization, machine translation, geocoded	Subscription only	Boolean and metadata query system (native)	Email alert
HealthMap	Google News, Moreover, ProMED, WHO, EuroSurveillance (4 languages)	Automatic	Categorization, geocoded, time coded, extra information	Open	Mapping, faceted browsing (native)	RSS feed
EpiSPIDER	ProMED, GDACS, CIA Factbook (English only)	Automatic	Categorization, geocoded, time coded, extra information	Open	Web exhibits, faceted browsing (imported)	RSS, JSON, KML feeds

*GPHIN, Global Public Health Intelligence Network; WHO, World Health Organization; RSS, Really Simple Syndication; EpiSPIDER, Semantic Processing and Integration of Distributed Electronic Resources for Epidemics (and disasters); GDACS, Global Disaster Alert Coordinating System; CIA, Central Intelligence Agency; JSON, JavaScript object notation; KML, keyhole markup language.

For completeness, the broadest range of sources is critical. GPHIN’s data comes from Factiva and Al Bawaba, which are subscription-only news aggregators. Their strategy is to rely on companies that sell the service of collecting event information from every pertinent news stream. In contrast, HealthMap’s strategy is to rely on open-access news aggregators (e.g., GoogleNews and Moreover) and curated sources (e.g., ProMED and EuroSurveillance). EpiSPIDER, until recently, has concentrated on curated sources only (e.g., ProMED, GDACS, and CIA Factbook). This distinction between free and paid sources raises the question of whether the systems have access to the same event information.

After the data sources have been chosen, the next step is to extract useful information among the incoming reports. First, at the level of the report stream, the system must filter out reports that are not disease related and categorize the remaining (disease-related) reports into predefined sets. Then, at a second level of triage, the information within each retrieved alert (e.g., an event’s location or reported disease) is assessed. GPHIN does this data characterization through automatic processing and human analysis, whereas HealthMap and EpiSPIDER rely mainly on automated techniques (although a person performs a daily scan of all HealthMap alerts and a sample of EpiSpider alerts).

After a report in the data stream is determined to be relevant, it is processed for dissemination. GPHIN automatically translates the reports into different languages and grants its clients access to the database through a custom search engine. GPHIN also decides which reports should be raised to the status of alerts and sent to its clients by email. HealthMap provides a geographic and temporal panorama of ongoing epidemics through an open-access user interface. It automatically filters out the reports that do not correspond to breaking alerts. The remaining alerts are prepared for display (timecodes and geocodes as well as disease category and data source) to allow faceted browsing and are linked to other information sources (e.g., the Wikipedia definition of the disease). These data are also provided as daily email digests to users interested in specific diseases and locations. Although GPHIN and HealthMap provide their own user interface, EpiSPIDER explores conventional formats for reports, adding time-coding, geo-coding, and country metadata for automatic integration with other information sources and versatile browsing by using existing open-source software. These reports are displayed under the name of Web Exhibits and include, for example, a mapping and a timeline view of the reports and a scatter plot of the alerts with respect to the originating country’s human development index and gross domestic product per capita.

A division arises between the HealthMap and EpiSPIDER strategies and the GPHIN strategy regarding the level of access granted to users. This division is due in part to the access policies of the data sources used by the systems, as discussed previously.

A discrepancy also exists in the amount of human expertise, and thus in the cost, required by the systems. These differences also raise the question of whether information from one system is more reliable than that of the others. Undertaking an evaluation of the systems in parallel is a critical next step. Also, all 3 systems are inherently prone to noise because most of the data sources they use or plan to use (Figure) for surveillance are not verified by public health professionals, so even if the system is supervised by a human analyst, it might still generate false alerts. False alerts need to be mitigated because they might have substantial undue economic and social consequences. Event-based disease surveillance may also benefit from algorithms linked by ontology (formal representation of a set of concepts within a domain and the relationships between those concepts) detecting precursors of disease events. Measurement and handling of input data’s reliability is a critical research direction.

Future development should focus on linking these systems more closely to public health practitioners in the field and establishing collaborative networks for alert verification and dissemination. Such development would ensure that event-based monitoring further establishes itself as an invaluable public health resource that provides critical context and an alternative to more traditional indicator-based outbreak reporting.

## References

[1] Feldmann H, Czub M, Jones S, Dick D, Garbutt M, Grolla A, Emerging and re-emerging infectious diseases. Med Microbiol Immunol (Berl). 2002;191:63–74. 10.1007/s00430-002-0122-5 12410344

[2] Lederberg J, Shope R, Oats S, eds. Emerging infections: microbial threats to health in the United States. Washington: National Academy Press; 1992. 25121245

[3] Wilson ME. Travel and the emergence of infectious diseases. Emerg Infect Dis. 1995;1:39–46. 8903157 10.3201/eid0102.950201 PMC2626831

[4] Morens DM, Folkers GK, Fauci AS. The challenge of emerging and re-emerging infectious diseases. Nature. 2004;430:242–9. 10.1038/nature02759 15241422 PMC7094993

[5] Mandl KD, Overhage JM, Wagner MM, Lober WB, Sebastiani P, Mostashari F, Implementing syndromic surveillance: a practical guide informed by the early experience. J Am Med Inform Assoc. 2004;11:141–50. 10.1197/jamia.M1356 14633933 PMC353021

[6] Chretien JP, Burkom HS, Sedyaningsih ER, Larasati RP, Lescano AG, Mundaca CC, Syndromic surveillance: adapting innovations to developing settings. PLoS Med. 2008;5:e72. 10.1371/journal.pmed.0050072 18366250 PMC2270304

[7] Chretien JP, Lewis SH. Electronic public health surveillance in developing settings: meeting summary. BMC Proc. 2008;2(Suppl 3):S1. 10.1186/1753-6561-2-s3-s1 PMC2587694 19025678

[8] Butler D. Disease surveillance needs a revolution. Nature. 2006;440:6–7. 10.1038/440006a 16511453

[9] Heymann DL, Rodier GR. Hot spots in a wired world: WHO surveillance of emerging and re-emerging infectious diseases. Lancet Infect Dis. 2001;1:345–53. 10.1016/S1473-3099(01)00148-7 11871807

[10] Morse SS. Global infectious disease surveillance and health intelligence. Health Aff (Millwood). 2007;26:1069–77. 10.1377/hlthaff.26.4.1069 17630449

[11] Woodall J. Official versus unofficial outbreak reporting through the Internet. Int J Med Inform. 1997;47:31–4. 10.1016/S1386-5056(97)00079-8 9506388

[12] Sturtevant JL, Anema A, Brownstein JS. The new international health regulations: considerations for global public health surveillance. Disaster Med Public Health Prep. 2007;1:117–21. 10.1097/DMP.0b013e318159cbae 18388639

[13] Grein TW, Kamara KB, Rodier G, Plant AJ, Bovier P, Ryan MJ. Rumors of disease in the global village: outbreak verification. Emerg Infect Dis. 2000;6:97–102. 10756142 10.3201/eid0602.000201 PMC2640857

[14] M’Ikanatha NM, Rohn DD, Robertson C, Tan CG, Holmes JH, Kunselman AR. Use of the Internet to enhance infectious disease surveillance and outbreak investigation. Biosecur Bioterror. 2006;4:293–300. 10.1089/bsp.2006.4.293 16999590

[15] Heymann DL, Rodier G. Global surveillance, national surveillance, and SARS. Emerg Infect Dis. 2004;10:173–5. 15040346 10.3201/eid1002.031038 PMC3322938

[16] Mawudeku A, Blench M. Global Public Health Intelligence Network (GPHIN). In: Proceedings of the 7th Conference of the Association for Machine Translation in the Americas 2006 [cited 2007 Apr 26]. Available from http://www.mt-archive.info/MTS-2005-Mawudeku.pdf

[17] Paquet C, Coulombier D, Kaiser R, Ciotti M. Epidemic intelligence: a new framework for strengthening disease surveillance in Europe. Euro Surveill. 2006;11:212–4. 17370970

[18] Madoff LC, Woodall JP. The Internet and the global monitoring of emerging diseases: lessons from the first 10 years of ProMED-mail. Arch Med Res. 2005;36:724–30. 10.1016/j.arcmed.2005.06.005 16216654

[19] Jones KE, Patel NG, Levy MA, Storeygard A, Balk D, Gittleman JL, Global trends in emerging infectious diseases. Nature. 2008;451:990–3. 10.1038/nature06536 18288193 PMC5960580

[20] Mawudeku A, Lemay R, Werker D, Andraghetti R, St. John R. The Global Public Health Intelligence Network. In: M’ikanatha NM, Lynfield R, Van Beneden CA, de Valk H, editors. Infectious disease surveillance, 1st ed. Lynn (MA): Blackwell Publishing; 2007

[21] Mykhalovskiy E, Weir L. The Global Public Health Intelligence Network and early warning outbreak detection: a Canadian contribution to global public health. Can J Public Health. 2006;97:42–4. 16512327 10.1007/BF03405213 PMC6976220

[22] Brownstein JS, Freifeld CC, Reis BY, Mandl KD. HealthMap: Internet-based emerging infectious disease intelligence. In: Infectious disease surveillance and detection: assessing the challenges—finding solutions. Washington: National Academy of Science; 2007. p. 183–204.

[23] Brownstein JS, Freifeld CC, Reis BY, Mandl KD. Surveillance sans frontières: Internet-based emerging infectious disease intelligence and the HealthMap project. PLoS Med. 2008;5:E151. 10.1371/journal.pmed.0050151 18613747 PMC2443186

[24] Hadhazy A. World wide wellness: online database keeps tabs on emerging health threats. Scientific American; July 8, 2008 [cited 2008 Dec 17]. Available from http://www.sciam.com/article.cfm?id=world-wide-wellness

[25] Larkin M. Technology and public health: HealthMap tracks global diseases. Lancet Infect Dis. 2007;7:91. 10.1016/S1473-3099(07)70018-X

[26] Captain S. Get your daily plague forecast. Wired News 2006 [cited 2008 Dec 17]. Available from http://www.wired.com/science/discoveries/news/2006/10/71961

[27] Rolka H, O’Connor JC, Walker D. Public health information fusion for situation awareness. Heidelberg: Springer Berlin; 2008.

[28] Waring SC, Brown BJ. The threat of communicable diseases following natural disasters: a public health response. Disaster Manag Response. 2005;3:41–7. 10.1016/j.dmr.2005.02.003 15829908

[29] Ivers LC, Ryan ET. Infectious diseases of severe weather-related and flood-related natural disasters. Curr Opin Infect Dis. 2006;19:408–14. 10.1097/01.qco.0000244044.85393.9e 16940862

[30] Fontelo P, Liu F, Ackerman M. askMEDLINE: a free-text, natural language query tool for MEDLINE/PubMed. BMC Med Inform Decis Mak. 2005;5:5. 10.1186/1472-6947-5-5 15760470 PMC1079856

